# Linking Gaming Disorder Tendencies in Children to Their Personality and Parental Gaming Behavior

**DOI:** 10.3389/fpsyt.2021.748195

**Published:** 2022-02-18

**Authors:** Jennifer Wernicke, Christian Montag

**Affiliations:** Department of Molecular Psychology, Institute of Psychology and Education, Ulm University, Ulm, Germany

**Keywords:** gaming, gaming disorder, personality, childhood, parental survey, neuroticism, conscientiousness

## Abstract

Playing computer and video games (so-called *gaming*) is of great popularity among children and adults. But for some people, gaming gets out of hand and can result in a *Gaming Disorder* (GD). Among others, high neuroticism and low conscientiousness are considered to play a role in the etiology of GD. Next to personality traits, environmental variables are debated such as the parental influence on children's gaming behavior. In detail, parental gaming behavior could have role model functions for children. Based on empirical observations and theoretical frameworks, it was hypothesized that higher tendencies to GD in children are associated with higher neuroticism, lower conscientiousness, and higher parental tendencies to GD. In an online survey *N* = 493 parents (*n* = 472 females; age: *M* = 36.30, *SD* = 5.46) rated their own and their children's (*n* = 233 girls, *n* = 260 boys; age: *M* = 6.03, SD = 2.30) tendencies to GD by the *Gaming Disorder Test* (GDT) and their children's personality by the *Big Five Inventory* (BFI). Neuroticism was significantly correlated with the GDT scores of boys (*rho* = 0.256, *p* = 0.001) and girls (*rho* = 0.300, *p* = 0.001), while a significant correlation with conscientiousness was only present for boys (*rho* = −0.196, *p* = 0.010). Parents' and children's general playing of computer and video games were interdependent [χ^2^(1) = 29.14, *p* < 0.001]; hence, if parents reported to be gamers, their children were more likely gamers as well. The GDT scores of parents and children were positively correlated (boys: *rho* = 0.36; *p* = 0.002; girls: *rho* = 0.33, *p* = 0.004). The results indicate that already in children the personality traits neuroticism and conscientiousness are associated with tendencies toward GD. Moreover, as gaming of parents and children was related to each other, it is conceivable that parents influence their children's gaming behavior via their own gaming behavior. Therefore, parents should be aware of their exemplary function for children and may overthink their own gaming behavior. But it has to be mentioned that the correlational nature of the present work allows no insights regarding causal relations.

## Introduction

Gaming is considered as playing online and/or offline computer and video games on a technical device such as computer, laptop, console, or smartphone (1), but excessive gaming can become a problematic behavior. Therefore, in May 2019, the World Health Organization (WHO) acknowledged Gaming Disorder (GD) as an official diagnosis in its recent 11th revision of the *International Classification of Diseases* (ICD-11) (2). The diagnosis criteria for online as well as offline GD are as follows: (i) impaired control over gaming, (ii) gaming becomes the main focus of life at cost of other everyday life activities, and (iii) gaming is continued, although the afflicted person already faces negative consequences in everyday functioning (family, school, work, etc.). It is important to note that a GD usually is diagnosed if the mentioned symptoms appear over a period of at least 12 months, with the exception of severe cases (2). According to our knowledge, no representative prevalence estimations of GD based on the ICD-11 diagnosis exist (3). However, a good indication represents the prevalence of Internet Gaming Disorder (IGD), a diagnosis of the *Diagnostic and Statistical Manual of Mental Disorders–5th edition* (DSM-5) (4), which is considered to be up to 3.05% worldwide, with males 2.5 times more often afflicted than females (5).

A prominent model to explain the development of GD (and other Internet and media-related use disorders) represents the I-PACE model (6). The model presumes that an *i*nteraction of certain predisposing *p*erson, *a*ffect, *c*ognition, and *e*xecution variables is of high relevance to understand the etiogenesis of GD. In the context of the present study, the main focus is on one important person variable mentioned within the I-PACE model, namely *personality* (6). Personality describes stable emotional, motivational, and cognitive dispositions of a person resulting in stable behavioral tendencies (7). In the context of the I-PACE model, among others, high neuroticism and low conscientiousness have been mentioned as relevant personality traits going along with higher tendencies toward addictive behaviors such as GD (6). The theoretical assumptions of the I-PACE model are supported by findings of systematic reviews that reported Internet-related GD being positively related with neuroticism but negatively related with conscientiousness (8, 9). A recent large-scale international study also provides support for these associations between personality and GD (10). However, the vast majority of research regarding Internet-related GD and personality was only conducted in adolescents and adults but not in children (8, 9). Nevertheless, playing computer and video games represents a popular leisure activity that is already performed at an early age (11, 12). However, several research works show that excessive gaming might have a negative impact on children's (psychosocial) development like on prosocial behavior, social competence, or academic engagement (12–14). Therefore, it is of special interest to get insights into vulnerability factors for exuberant gaming or even a GD in children, and high neuroticism and low conscientiousness could be two such factors.

Especially in young children, not only their own personality but also their parents might influence their gaming behavior. Within a meta-analysis, it was shown that the parent–child relationship as well as the parental influence on gaming are of relevance whether children and adolescents develop a problematic gaming behavior (15). While a close parent–child relationship (16) and parental supervision of gaming (17) might have a protective influence, parental gaming is considered to be a risk factor (18, 19). The latter might be explained by the *Social Learning Theory* (20, 21) according to which children tend to imitate their role models' behavior as this is especially appealing for them. That leads to the assumption that next to children's personality, parental gaming might be another vulnerability factor for children to develop a problematic gaming behavior or even a GD.

Therefore, the aim of the present work was to investigate two possible vulnerability factors for problematic gaming behavior in children: children's personality and parental gaming. It was expected that in children, the same associations between the personality traits neuroticism and conscientiousness and tendencies to GD can be observed as in adolescents and adults. Furthermore, it was expected that parental and children's gaming are positively associated with each other. To sum up, the following three hypotheses are investigated in the present study:

i) Higher neuroticism and lower conscientiousness are linked to higher GD tendencies in children.

ii) Parents reporting to play computer and video games are more likely to have children who play computer and video games as well.

iii) Tendencies to GD of parents and their children are positively correlated with each other.

It has to be mentioned that the present work is part of a larger project, and first results were already presented in Wernicke and Montag (22) to the point when data of 249 participants were available. In this subsample, the authors observed that parents who reported to play computer and video games were more likely to report that their children were gaming as well. Furthermore, the GD tendencies of parents and their children were positively associated with each other (22). Please note that this work is available in German language only.

## Materials and Methods

### Procedure

Data were collected *via* a two-part parental online survey between February 2020 and June 2021. The study was advertised in schools, kindergartens, an online platform of pediatricians, and *via* different online media channels (Facebook, Instagram). In the first part of the survey, children's personality and gaming behavior were assessed; in the second part, parents' gaming behavior was of interest. Participation was anonymous. All participants provided electronic consent before filling in the survey. The present study was approved by the local Ethics Committee of Ulm University, Ulm, Germany, in August 2018 (253/18).

### Participants

In total, *N* = 493 German parents (19 males, 472 females, 2 neither defining as male nor female; age in years: *M* = 36.30, *SD* = 5.46) participated in the present study. Inclusion criteria were a child's minimum age of 3 years, available data for both parts of the survey, and no double participation. Double participation was checked with a yes/no item “Did you participate in this study before?”. If former participation was stated with “yes”, the date of former participation was asked. If a date different from the recruiting phase of the study (February 2020 to June 2021) was stated, participants were included in the data analyses. Every parent filled in the survey for only one child. Parents of several children could decide themselves for which child they fill in the survey. Therefore, data of *N* = 493 children were available (260 boys, 233 girls; age in years: *M* = 6.03, *SD* = 2.30).

As mentioned earlier, the data of *n* = 249 parents and their children were already included in the paper by Wernicke and Montag (22). In the meantime, a larger sample was collected. As research questions here overlap only partly with the former work, these *n* = 249 data sets are also included in the present work. Therefore, results are reported only for the total sample of *N* = 493.

### Gaming Behavior

It was separately asked for children (external report) and parents (self-report) if online and/or offline video/computer games were played at least occasionally within the last 12 months (yes/no question). That was the case for *n* = 195 (39.6%) parents and for *n* = 247 (50.1%) children. The overlap of gaming parents and gaming children within the same family was *n* = 127. Tendencies toward GD were assessed by the *Gaming Disorder Test* (GDT) (1). Parents reported their own tendencies via self-report in the German version of the GDT as presented in Montag et al. (23). For gaming children (*n* = 247), an external report with a German modified version of the GDT suitable for parents was used (22). Both versions of the GDT consist of four items that are answered on a five-point Likert scale (1 = “never” to 5 = “very often”). Sum scores were calculated separately for the self-report (parents' GDT; α = 0.78) and the external report (child's GDT; α = 0.80). A confirmatory factor analysis (CFA) with maximum likelihood (ML) estimators was performed to assess the model fit of both GDT versions. For the external GDT (child's gaming behavior), the model fit was not acceptable [χ^2^(2) = 83.191, *p* < 0.001; CFI = 0.817; TLI = 0.451; RMSEA = 0.405; SRMR = 0.102], but the factor loadings of all items were acceptable (λ_1_ = 0.812, λ_2_ = 0.869, λ_3_ = 0.683, λ_4_ = 0.552) and in accordance with the reported loadings by Pontes et al. (1). The model fit for the self-report (parents' GDT) was in the lower range of acceptability [χ^2^(2) = 19.959, *p* < 0.001; CFI = 0.934; TLI = 0.802; RMSEA = 0.215; SRMR = 0.049], with factor loadings of λ_1_ = 0.687, λ_2_ = 0.774, λ_3_ = 0.779, and λ_4_ = 0.662.

### Personality

Children's personality was assessed as an external report. To do so, a version of the *Big Five Inventory* (BFI) by John and Srivastava (24) for parents to describe their children's personality was used. Here, a German version of this questionnaire was applied. For this, the English version of the parental BFI was translated into German and independently back-translated into English. The German translation was oriented toward the German BFI self-report by Rammstedt and Danner (25). The parental BFI consists of 46 items that are answered on a five-point Likert scale (1 = “disagree strongly” to 5 = “agree strongly”). Mean scores were calculated for the five personality dimensions extraversion (8 items, α = 0.81), agreeableness (9 items, α = 0.75), conscientiousness (9 items, α = 0.88), neuroticism (8 items, α = 0.83), and openness to experiences (10 items, α = 0.75). The two remaining items of the parental BFI measure the scale liking, which was not of relevance for the present work. A CFA with an ML estimator was performed. The model fit for the proposed factor structure of the parental BFI was in the lower range of acceptability [χ ^2^(892) = 2,871.060, *p* < 0.001; CFI = 0.763; TLI = 0.748; RMSEA = 0.067; SRMR = 0.091].

### Control Variables

Parents' age as well as children's age and gender were considered as control variables. It was not possible to control for parents' gender due to the high number of participating mothers.

### Statistical Analyses

All variables of interest were checked for normal distribution by Shapiro–Wilk tests. As none of the variables followed a normal distribution (see Table 1), only non-parametric tests were performed. Tests for control variables were Spearman rank correlations, a χ^2^ test of independence, and Mann–Whitney *U*-tests. All significances were tested two-tailed. As parent's age, children's age, as well as children's gender were associated with some variables of interest, these variables were controlled in further analyses if this was possible. Associations between children's personality and GDT scores were analyzed *via* partial Spearman rank correlations (controlled for children's age), separately for boys and girls and for reasons of completeness also for boys and girls together. Given the directed hypothesis, correlations with neuroticism and conscientiousness were performed one-tailed; correlations with extraversion, agreeableness, and openness to experiences were performed two-tailed instead. With χ^2^ tests of independence, it was investigated if general parental and childhood gaming were independent of each other (two-tailed tested, no control for age and gender possible). While doing so, one χ^2^ test was conducted in the independent replication subsample of *n* = 244, which was not included in Wernicke and Montag (22). Another χ^2^ test was conducted in the total sample of *N* = 493. Finally, to check for associations between parents' and children's tendencies toward GD partial Spearman rank correlations were performed separately for boys and girls and for reasons of completeness also for boys and girls together (controlled for parents' and children's age; one-tailed tested). Again, correlation analyses were performed twice: once in the independent replication subsample, which was not included in Wernicke and Montag (22), and once in the total sample. As for these correlation analyses solely parents and children who were playing video/computer games were of relevance, the respective subsamples are smaller than in the remaining analyses.

**Table 1:** Descriptive statistics of variables of interest.

	**M**	**SD**	**min**	**max**	**SW** **statistics**	***df***	***P***
**Parents (*****N =*** **493)**							
Age	36.30	5.46	23	65	0.962	493	<0.001
GDT (*n* = 195)	5.46	2.11	4	20	0.703	195	<0.001
**Children (*****N*** **= 493)**							
Age	6.03	2.30	3	13	0.932	493	<0.001
GDT (*n* = 247)	6.74	2.99	4	20	0.840	247	<0.001
Extraversion	3.83	0.71	1.50	5.00	0.964	493	<0.001
Agreeableness	3.77	0.57	1.78	5.00	0.973	493	<0.001
Conscientiousness	3.30	0.77	1.11	5.00	0.984	493	<0.001
Neuroticism	2.77	0.71	1.25	4.75	0.985	493	<0.001
Openness to experiences	4.02	0.54	1.80	5.00	0.946	493	<0.001

*M, mean; SD, standard deviation; min, minimum; max, maximum; SW, Shapiro–Wilk; df, degrees of freedom; GDT, Gaming Disorder Test*.

A general alpha level of 0.05 was accepted. If necessary, this alpha level was adjusted by Bonferroni correction for multiple testing (see results for detailed information). All statistical analyses were conducted using SPSS 26 with the exception of the CFAs that were performed in R using the package lavaan (26). Please note that power analyses were not conducted because it was not clear what effect sizes to expect due to limited studies investigating tendencies toward GD and personality in children.

## Results

### Descriptive Statistics

Descriptive statistics are presented separately for parents and children in Table 1.

### Influence of Age and Gender on Gaming

Parents' age correlated significantly negatively with the parental GDT score (*rho* = −0.156, *p* = 0.030). Children playing computer and video games had older parents (*U* = 23267.50, *p* < 0.001, *M*_*gamer*_ = 37.43, *M*_*non*–*gamer*_ = 35.17) and were older themselves (*U* = 16557.00, *p* < 0.001, *M*_*gamer*_ = 6.93, *M*_*non*–*gamer*_ = 5.13) than children who were not gaming. Furthermore, children's age correlated significantly with children's GDT score (*rho* = 0.141, *p* = 0.027), with extraversion (*rho* = −0.104, *p* = 0.021), and with neuroticism (*rho* = 0.101, *p* = 0.025).

More boys than girls were playing computer and video games [gaming boys vs. girls: *n* = 142 to *n* = 105; non-gaming boys vs. girls: *n* = 118 to *n* = 128; χ^2^(1) = 4.48, *p* = 0.034]. Boys had also significantly higher GDT scores than girls. On the contrary, boys had significantly lower scores for agreeableness, conscientiousness, and openness to experiences than girls. After Bonferroni correction for multiple testing to α of 0.007 (0.05/7), the gender difference for agreeableness was not statistically significant anymore. Results of Mann–Whitney *U*-tests regarding gender differences are presented in Table 2.

**Table 2:** Mann–Whitney *U*-tests to check for gender differences in children.

	**Gender**	***M (SD)***	**Mann–** **Whitney *U***
			***P* (two-** **tailed)**
Age child	Male	6.20 (2.35)	27775.50
	Female	5.85 (2.24)	0.108
GDT child	Male^a^	7.63 (3.27)	4366.50
	Female^b^	5.55 (2.02)	<0.001
Extraversion	Male	3.79 (0.70)	27939.00
	Female	3.87 (0.72)	0.136
Agreeableness	Male	3.70 (0.59)	26449.00
	Female	3.84 (0.52)	0.015
Conscientiousness	Male	3.18 (0.74)	24151.00
	Female	3.43 (0.77)	<0.001
Neuroticism	Male	2.82 (0.72)	27682.00
	Female	2.71 (0.69)	0.098
Openness to experiences	Male	3.94 (0.57)	24425.00
	Female	4.12 (0.50)	<0.001

*M, mean; SD, standard deviation; n(boys), 260; n(girls), 233; GDT, Gaming Disorder Test*.

a *n(gaming boys), 142*;

b *n(gaming girls), 105*.

### Gaming and Personality in Children

As hypothesized, within the total sample of playing children (boys and girls were analyzed together), the GDT score was significantly positively correlated with neuroticism but significantly negatively correlated with conscientiousness. Moreover, associations between the GDT score and extraversion, agreeableness, and openness to experiences were exploratory analyzed. These three personality dimensions were all significantly negatively correlated with the GDT score.

Regarding gender differences, for boys, the GDT score was significantly positively correlated with neuroticism but significantly negatively correlated with conscientiousness, extraversion, agreeableness, and openness to experiences. For girls, the correlation between the GDT score and neuroticism was significantly positive. The remaining correlations were negatively directed but were not statistically significant. After Bonferroni correction for multiple testing to α of 0.0033 (0.05/15), most of the correlations remain statistically significant. All correlations of the GDT score with personality traits are presented in detail in Table 3.

**Table 3:** Partial Spearman rank correlations of GDT scores and personality in children who are gaming.

	**Total (*****n*** **= 247)**		**Boys (*****n*** **= 142)**		**Girls (*****n*** **= 105)**	
	***rho***	***P***	***rho***	***P***	***rho***	***P***
Conscientiousness ^a^	**−0.259**	<0.001	– 0.196	0.010	–0.143	0.074
Neuroticism^a^	**0.323**	<0.001	**0.256**	0.001	**0.300**	0.001
Extraversion^b^	**−0.189**	0.003	–0.170	0.044	–0.137	0.166
Agreeableness^b^	**−0.249**	<0.001	**−0.252**	0.003	–0.148	0.133
Openness to experiences^b^	**−0.210**	<0.001	–0.237	0.005	– 0.054	0.589

*GDT, Gaming Disorder Test. All correlations are controlled for children's age*.

a *Correlations were one-tailed tested*.

b *Correlations were two-tailed tested. Correlations printed in bold are statistically significant after Bonferroni correction. The alpha level was corrected to 0.0033 (0.05/15) as a total of fifteen correlations with children's GDT score were performed*.

### Parental and Childhood Gaming

Whether parents and children were gamers or non-gamers was dependent of each other in the total sample of N = 493 [χ^2^(1) = 29.14, *p* < 0.001] as well as in the independent replication subsample of *n* = 244 [χ^2^(1) = 13.77, *p* < 0.001]. In detail, the χ^2^ tests of independence showed that more children were gamers when their parents were gamers, too, and vice versa, more children were non-gamers when their parents were non-gamers as well (for more details regarding the total sample see Table 4).

**Table 4:** Cross-table of observed vs. expected frequencies of gamers and non-gamers among parents and children, total sample (*N* = 493).

			**Parents**		**Σ**
			**Gaming**	**No gaming**	
Children	Gaming	Observed	**127**	120	247
		Expected	97.7	149.3	247.0
	No gaming	Observed	68	**178**	246
		Expected	97.3	148.7	246.0
	Σ	Observed	195	298	493
		Expected	195.0	298.0	493.0

*Numbers printed in bold represent the actual overlap of gaming children and gaming parents as well as non-gaming children and non-gaming parents*.

Furthermore, when considering the total sample the GDT scores of gaming parents and of their gaming children were positively correlated (*n* = 127: *rho* = 0.33, *p* < 0.001). This was also the case when performing the correlation analyses separately for gaming boys (*n* = 63: *rho* = 0.36; *p* = 0.002) and gaming girls (*n* = 64: *rho* = 0.33, *p* = 0.004).

In the independent replication subsample the GDT scores of gaming parents and of their gaming children were also positively correlated (*n* = 67: *rho* = 0.29, *p* = 0.010). This was also the case when correlation analyses were performed separately for gaming boys (*n* = 29: *rho* = 0.45, *p* = 0.010) and gaming girls (*n* = 38, *rho* = 0.36, *p* = 0.016). After Bonferroni correction for multiple testing to alpha of 0.008 (0.05/6), correlations of the independent replication subsample were not statistically significant.

## Discussion

The present study investigated the associations between children's tendencies toward GD and their own personality traits as well as their parents' gaming behavior.

Regarding personality, it was hypothesized that GD tendencies in children are positively correlated with neuroticism but negatively correlated with conscientiousness. This hypothesis is supported by the data of the current study. Therefore, the found results provide support that the personality traits neuroticism and conscientiousness are of relevance for tendencies toward GD not only in adolescents and adults (6, 8) but also in children.

Neuroticism is described as emotional instability with a person reacting with negative emotions such as anxiety, anger, or sadness to stressful events (27). It is assumed that people with high neuroticism use gaming as a maladaptive coping strategy to regulate their negative emotions and/or to deal with negative life events (8). Gaming might represent a distraction from negative emotions already in children and therefore be a vulnerability factor for GD already at an early age.

Conscientiousness describes a person to have high self-control, accuracy, and self-determination (28). Especially a lack of self-control might be of relevance for longer and more excessive gaming. But we want to stress that the observed expressions of personality within the present sample are not pathological but in the range of normality. Moreover, especially in children, personality traits change during development, and self-control is developed within late adolescence and early adulthood that results in higher conscientiousness (29). Therefore, the connection between personality and gaming in childhood in our study only represents a snapshot, and a reduction of tendencies toward GD due to better self-regulation strategies in later childhood is possible.

Interestingly, the positive association between GD tendencies and neuroticism was present in both genders when analyses were conducted separately for boys and girls. For the association with conscientiousness only in boys, a statistically significant correlation was present, while for girls, a non-significant trend in the same direction existed. The lack of significance in the girl sample might be due to two aspects. First, the girls' sample was smaller than the boys' sample. Second, girls had lower GDT scores but higher conscientiousness scores than boys, wherefore detecting an effect is more difficult in the girls' sample compared to the boys' sample; especially as the variance within the GDT scores of girls was smaller than in boys. Perhaps the higher conscientiousness scores reflect an earlier maturation process of girls compared to boys, especially as girls were even half a year younger than boys in the present total sample.

Regarding personality, also significantly negative associations between GD tendencies and the personality traits extraversion, agreeableness, and openness to experiences could be observed. As we did not hypothesize these associations, we do not want to overinterpret our findings, but are interested to see if these observations can also be made in future scientific works investigating links between personality and GD in childhood. From what we observe in the present work, children with tendencies toward GD are rated by their parents as being less curious regarding their environment (lower openness to experience), less empathic and cooperative (lower agreeableness), and more introverted (lower extraversion). As the present study is of correlative nature, it is not possible to determine if these manifestations of personality traits are a result of excessive gaming or if these provide a disposition toward GD. In line with the I-PACE model, we would expect the latter to be true (6); hence, certain constellations of personality traits might make a person more vulnerable toward GD than others. Nevertheless, it is important to keep in mind that personality in children is not necessarily stable but is rather prone to change during development (29–31). Especially agreeableness and conscientiousness are supposed to increase between early childhood and the first years of elementary school (32).

Moreover, even though personality is of relevance to understand the etiogenesis of GD, it is only one relevant factor among many. In the present work, we therefore aimed to shed light on a further factor, namely parental gaming, while we especially focused on the relationship between parental and childhood gaming. We hypothesized parental and childhood gaming to be interdependent. The results support this hypothesis as gaming parents were more likely to report that their children are gaming as well. Additionally, the GDT scores of parents and their children were positively correlated with each other. The results replicate the findings by Wernicke and Montag (22) and other research according to which parental gaming is related to children's gaming behavior (18, 19). Even if in the present study no statements regarding causality can be made, also on the background of *Social Learning Theory* (20, 21), it seems more likely that parents' gaming influences children's gaming instead of vice versa. Children are good observers and copy the behavior of their environment and obviously also the behavior of their parents. Therefore, parents should reflect on their own gaming behavior and consider reducing it to prevent their children from developing a problematic gaming behavior or even a GD.

One strength of the present study is the investigation of three potential risk factors that might be related to children's tendencies to GD, namely high neuroticism, low conscientiousness, and parental gaming. Actually, these hypothesized relations were supported by the findings of the present work. This is an important contribution in understanding the etiology of GD. Furthermore, the replication of the findings by Wernicke and Montag (22) regarding the positive association between parental and childhood gaming is another strength, as independent replications are highly relevant.

But the present study also has some shortcomings. One limiting factor is that frequency and duration of gaming were not assessed, neither for children nor for parents. However, the amount of time spent gaming is not an indicator for GD but clearly accompanies a problematic gaming behavior (2). Moreover, recent work demonstrates how difficult it is to assess the time spent on technology use *via* self-report (33). Another limitation is the homogenous parental sample as a vast majority of participating parents were mothers. In future research works, it would be of interest to examine whether the association between parental and childhood gaming differs depending on whether fathers or mothers are investigated. Limitations regarding the interpretation of results also arise from the external report of children's gaming behavior and personality and the self-report of parents' gaming. In this realm, it has to be mentioned that some researchers propose to assess temperament instead of personality in children (34). But personality develops from temperament (34). Also temperament and personality in children are reported to be rather similar (35, 36) and to be at least moderately related with each other (37). Therefore, we considered the assessment of personality traits to be appropriate. Finally, due to the study design, no statements regarding causality are possible.

## Conclusion

Gaming—hence playing computer and video games—is already popular in young children. But there is a risk that children develop an excessive or problematic gaming behavior. Therefore, the present study investigated the relationship between tendencies to GD and two of its possible vulnerability factors: children's personality traits and parental gaming. As in adolescents and adults, higher neuroticism and lower conscientiousness were related to higher GD tendencies in children. Additionally, associations of higher tendencies towards GD with lower extraversion, agreeableness, and openness to experiences were present. But these links of personality traits and GD need to be further investigated regarding their robustness in childhood as these relations were not hypothesized in the present work. Moreover, gaming behavior of parents was positively associated with children's gaming, too. The findings of the study add to the literature that already in children relations between personality traits and gaming behavior exist and that parents' gaming behavior can be a vulnerability factor for disordered gaming in children. In the future, research projects with a longitudinal design are of importance to understand the causality behind the reported findings.

## Data Availability Statement

The raw data supporting the conclusions of this article is provided here: osf.io/ev93y.

## Ethics Statement

The studies involving human participants were reviewed and approved by Ethics Committee of Ulm University, Ulm, Germany. Written informed consent for participation was not required for this study in accordance with the national legislation and the institutional requirements.

## Author Contributions

JW and CM designed the present study and drafted the present manuscript. JW conducted the data collection and performed the statistical analyses. The final version of the manuscript was approved by both authors.

## Conflict of Interest

The authors declare that the research was conducted in the absence of any commercial or financial relationships that could be construed as a potential conflict of interest.

## Publisher's Note

All claims expressed in this article are solely those of the authors and do not necessarily represent those of their affiliated organizations, or those of the publisher, the editors and the reviewers. Any product that may be evaluated in this article, or claim that may be made by its manufacturer, is not guaranteed or endorsed by the publisher.
